# 2-Trifluoro­methyl-1*H*-benzimidazol-3-ium nitrate

**DOI:** 10.1107/S1600536812009415

**Published:** 2012-03-10

**Authors:** Ming-Liang Liu

**Affiliations:** a Ordered Matter Science Research Center, Southeast University, Nanjing 211189, People’s Republic of China

## Abstract

Ihe title salt, C_8_H_6_F_3_N_2_^+^·NO_3_^−^, the F atoms of the triflouromethyl group are disordered over two sets of sites in a 0.58 (2):0.42 (2) ratio. In the crystal, N—H⋯O hydrogen bonds link the cations and anions into chains running parallel to the *b* axis. There is π–π stacking between symmetry-related benzene rings with a centroid-centroid distance of 3.949 (3) Å. The crystal studied was a non-merohedral twin, with a 19% minor component.

## Related literature

The title compound was synthesized as part of a search for potential ferroelectric compouns. For background to ferroelectric complexes, see: Fu *et al.* (2011 ▶); Zhang *et al.* (2010 ▶). For related structures, see: Liu (2011*a* ▶,*b* ▶, 2012 ▶). For the separation of the non-merohedrally twinned diffraction data, see: Spek (2009 ▶).
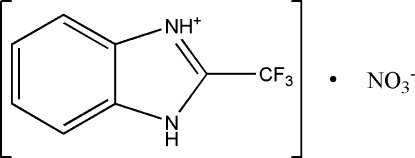


## Experimental

### 

#### Crystal data

C_8_H_6_F_3_N_2_^+^·NO_3_^−^
*M* *_r_* = 249.16
Triclinic, 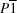

*a* = 7.2745 (15) Å
*b* = 9.0962 (18) Å
*c* = 9.4502 (19) Å
α = 61.53 (3)°
β = 71.18 (3)°
γ = 82.41 (3)°
*V* = 520.1 (3) Å^3^
*Z* = 2
Mo *K*α radiation
μ = 0.16 mm^−1^
*T* = 293 K
0.20 × 0.20 × 0.20 mm

#### Data collection

Rigaku SCXmini diffractometer
Absorption correction: multi-scan (*CrystalClear*; Rigaku, 2005 ▶) *T*_min_ = 0.969, *T*_max_ = 0.969
1828 measured reflections
1828 independent reflections
910 reflections with *I* > 2σ(*I*)

#### Refinement

*R*[*F* ^2^ > 2σ(*F* ^2^)] = 0.072
*wR*(*F* ^2^) = 0.227
*S* = 1.01
1828 reflections
183 parameters
H-atom parameters constrained
Δρ_max_ = 0.23 e Å^−3^
Δρ_min_ = −0.27 e Å^−3^

### 

Data collection: *CrystalClear* (Rigaku, 2005 ▶); cell refinement: *CrystalClear*; data reduction: *CrystalClear*; program(s) used to solve structure: *SHELXS97* (Sheldrick, 2008 ▶); program(s) used to refine structure: *SHELXL97* (Sheldrick, 2008 ▶); molecular graphics: *PLATON* (Spek, 2009) ▶; software used to prepare material for publication: *SHELXTL* (Sheldrick, 2008 ▶).

## Supplementary Material

Crystal structure: contains datablock(s) I, global. DOI: 10.1107/S1600536812009415/go2046sup1.cif

Structure factors: contains datablock(s) I. DOI: 10.1107/S1600536812009415/go2046Isup2.hkl

Supplementary material file. DOI: 10.1107/S1600536812009415/go2046Isup3.cml

Additional supplementary materials:  crystallographic information; 3D view; checkCIF report

## Figures and Tables

**Table 1:** Hydrogen-bond geometry (Å, °)

*D*—H⋯*A*	*D*—H	H⋯*A*	*D*⋯*A*	*D*—H⋯*A*
N2—H2*A*⋯O1	0.86	1.86	2.703 (5)	168
N3—H3*A*⋯O2^i^	0.86	1.84	2.682 (5)	165

Symmetry code: (i) 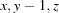.

**Fig. 1.**
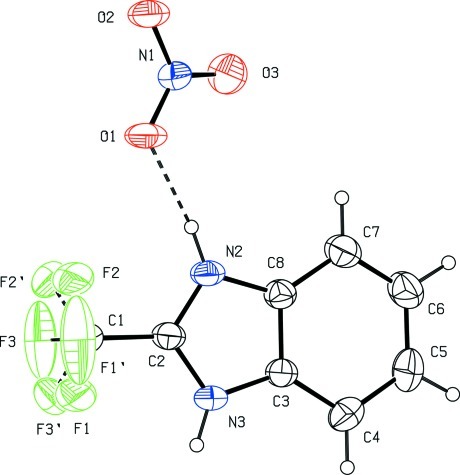
The molecular structure of the title compound, showing the atomic numbering scheme with 30% probability displacement ellipsoids.

**Fig. 2.**
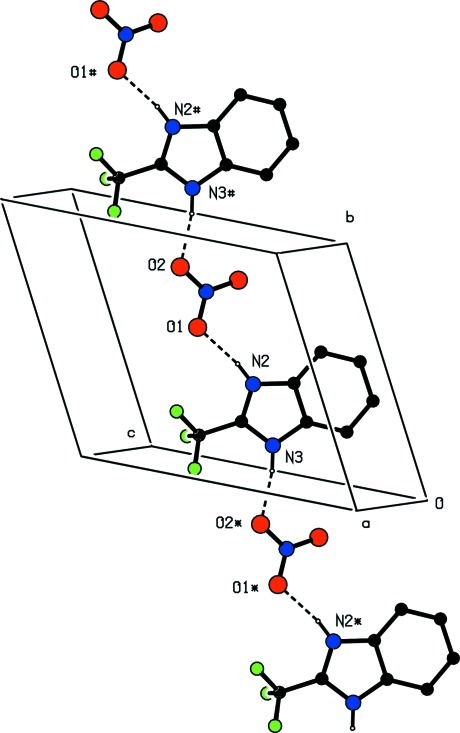
View of the cation/anion chain running parallel to the b axis. Minor component F atoms and H atoms not involved in the hydrogen bonding are omitted for clarity. Atoms labelled with a *(star) are at (x,-1+y,z) and those labelled with a #(hash) are at (x,1+y,z).

## supplementary crystallographic information

### Comment

Recently much attention has been devoted to crystals containing organic ions and inorganic ions due to the possibility of tuning their special structural features and their potential ferroelectrics properties (Fu *et al.*, 2011; Zhang *et al.*, 2010.). In our laboratory, the title compound (I) has been synthesized to investigate its potential ferroelectric propeties. However, it was found that the dielectric constant of the compound as a function of temperature indicated that the permittivity is basically temperature-independent (*ε* = C/(T–T_0_)), suggesting that this compound is not ferroelectric or there may be no distinct phase transition occurring within the measured temperature range (below the melting point).

The asymmetric unit that consists of one 2-trifluoromethyl-1H-benzimidazole cation and nitrate anionwhich are linked by N2—H2A···O1 hydrogen bond. Figure 1. The N3—H3A···O2 hydrogen bond links the the asymmetric units together into chains which run parallel to the b-axis. (Fig 2). There is π···π stacking between the six-membered rings at (x,y,z) and (1-x,1-y,-z) with a centroid to centroid distance of 3.949 (3)Å, perpendicular distance between the planes of 3.514 (2)Å and a slippage of 1.802Å. The triflouromethyl group is disordered.

### Experimental

0.144 g (1 mmol) of 2-trifluoromethyl-1*H*-benzimidazol was firstly dissolved in 30 ml of ethanol, to which 0.063 g (1 mmol) of nitric acid was added forming a solution at the ambient temperature. Single crystals suitable for X-ray structure analysis were obtained by the slow evaporation of the above solution after 3 days in air.

### Refinement

H atoms were placed in calculated positions (N—H = 0.89 Å; C—H = 0.93Å for C*sp^2^* atoms and C—H = 0.96Å and 0.97Å for C*sp^3^* atoms), assigned fixed *U*_iso_ values [*U*_iso_ = 1.2*U*eq(C*sp^2^*) and 1.5*U*eq(C*sp^3^*,*N*)] and allowed to ride. The trifluoromethyl group is modelled as being disordered over two sites with refined site occupancies of 0.58 and 0.42 respectively. The crystal was twinned. A .hkl file suitable for twin refinement was created using the TwinRotMat option in PLATON (Spek, 2009), and refined using the HKLF 5 option in SHELXL (Sheldrick,2008), giving a final BASF value of 0.19. Thus the ratio of the twin components was 0.81/0.19.

### Crystal data

**Table:**

C_8_H_6_F_3_N_2_^+^·NO_3_^−^	*V* = 520.1 (3) Å^3^
*M**_r_* = 249.16	*Z* = 2
Triclinic, *P*1	*F*(000) = 252
Hall symbol: -P 1	*D*_x_ = 1.591 Mg m^−^^3^
*a* = 7.2745 (15) Å	Mo *K*α radiation, λ = 0.71073 Å
*b* = 9.0962 (18) Å	θ = 3.3–25.0°
*c* = 9.4502 (19) Å	µ = 0.16 mm^−^^1^
α = 61.53 (3)°	*T* = 293 K
β = 71.18 (3)°	Block, colourless
γ = 82.41 (3)°	0.20 × 0.20 × 0.20 mm

### Data collection

**Table:**

Rigaku SCXmini diffractometer	1828 independent reflections
Radiation source: fine-focus sealed tube	910 reflections with *I* > 2σ(*I*)
Graphite monochromator	*R*_int_ = 0.000
CCD_Profile_fitting scans	θ_max_ = 25.0°, θ_min_ = 3.3°
Absorption correction: multi-scan (*CrystalClear*; Rigaku, 2005)	*h* = −8→8
*T*_min_ = 0.969, *T*_max_ = 0.969	*k* = −10→10
1828 measured reflections	*l* = −9→11

### Refinement

**Table:**

Refinement on *F*^2^	Primary atom site location: structure-invariant direct methods
Least-squares matrix: full	Secondary atom site location: difference Fourier map
*R*[*F*^2^ > 2σ(*F*^2^)] = 0.072	Hydrogen site location: inferred from neighbouring sites
*wR*(*F*^2^) = 0.227	H-atom parameters constrained
*S* = 1.01	*w* = 1/[σ^2^(*F*_o_^2^) + (0.1061*P*)^2^] where *P* = (*F*_o_^2^ + 2*F*_c_^2^)/3
1828 reflections	(Δ/σ)_max_ < 0.001
183 parameters	Δρ_max_ = 0.23 e Å^−^^3^
0 restraints	Δρ_min_ = −0.27 e Å^−^^3^

### Special details

**Table:**

Geometry. All esds (except the esd in the dihedral angle between two l.s. planes) are estimated using the full covariance matrix. The cell esds are taken into account individually in the estimation of esds in distances, angles and torsion angles; correlations between esds in cell parameters are only used when they are defined by crystal symmetry. An approximate (isotropic) treatment of cell esds is used for estimating esds involving l.s. planes.
Refinement. Refinement of F^2^ against ALL reflections. The weighted R-factor wR and goodness of fit S are based on F^2^, conventional R-factors R are based on F, with F set to zero for negative F^2^. The threshold expression of F^2^ > 2sigma(F^2^) is used only for calculating R-factors(gt) etc. and is not relevant to the choice of reflections for refinement. R-factors based on F^2^ are statistically about twice as large as those based on F, and R- factors based on ALL data will be even larger.

### Fractional atomic coordinates and isotropic or equivalent isotropic displacement parameters (Å^2^)

**Table:**

	*x*	*y*	*z*	*U*_iso_*/*U*_eq_	Occ. (<1)
N2	0.2477 (5)	0.3645 (4)	0.4631 (4)	0.0551 (10)	
H2A	0.2479	0.4361	0.4981	0.066*	
O2	0.2968 (6)	0.7956 (4)	0.5882 (4)	0.0824 (12)	
O1	0.2843 (6)	0.5588 (4)	0.5979 (5)	0.0849 (12)	
N3	0.2467 (5)	0.1269 (4)	0.4602 (4)	0.0541 (10)	
H3A	0.2470	0.0209	0.4932	0.065*	
N1	0.2547 (6)	0.7095 (5)	0.5303 (5)	0.0586 (11)	
O3	0.1883 (5)	0.7778 (5)	0.4106 (5)	0.0839 (12)	
F1	0.343 (3)	−0.0238 (12)	0.7647 (14)	0.104 (7)	0.58 (2)
F2	0.331 (4)	0.1984 (11)	0.7662 (17)	0.108 (7)	0.58 (2)
F3	0.0708 (17)	0.077 (4)	0.8311 (11)	0.166 (11)	0.58 (2)
F1'	0.396 (2)	0.077 (7)	0.7605 (16)	0.187 (17)	0.42 (2)
F2'	0.144 (5)	0.1946 (18)	0.8128 (15)	0.110 (8)	0.42 (2)
F3'	0.132 (4)	−0.0270 (19)	0.8115 (17)	0.107 (8)	0.42 (2)
C2	0.2467 (6)	0.2011 (5)	0.5524 (6)	0.0531 (12)	
C3	0.2462 (7)	0.2518 (5)	0.3017 (5)	0.0523 (12)	
C8	0.2483 (6)	0.4019 (5)	0.3024 (5)	0.0520 (12)	
C7	0.2462 (7)	0.5527 (6)	0.1610 (6)	0.0681 (14)	
H7	0.2462	0.6545	0.1619	0.082*	
C5	0.2441 (8)	0.3936 (8)	0.0199 (6)	0.0781 (16)	
H5	0.2447	0.3947	−0.0791	0.094*	
C6	0.2442 (8)	0.5454 (7)	0.0225 (7)	0.0778 (16)	
H6	0.2428	0.6445	−0.0741	0.093*	
C1	0.2396 (12)	0.1092 (7)	0.7352 (7)	0.0659 (14)	
C4	0.2431 (8)	0.2420 (7)	0.1588 (6)	0.0698 (15)	
H4	0.2405	0.1405	0.1580	0.084*	

### Atomic displacement parameters (Å^2^)

**Table:**

	*U*^11^	*U*^22^	*U*^33^	*U*^12^	*U*^13^	*U*^23^
N2	0.073 (3)	0.041 (2)	0.063 (2)	0.0001 (18)	−0.026 (2)	−0.029 (2)
O2	0.130 (3)	0.050 (2)	0.090 (3)	0.009 (2)	−0.050 (2)	−0.041 (2)
O1	0.140 (3)	0.042 (2)	0.093 (3)	0.010 (2)	−0.050 (2)	−0.0389 (19)
N3	0.071 (3)	0.038 (2)	0.060 (2)	−0.0001 (17)	−0.019 (2)	−0.0276 (19)
N1	0.071 (3)	0.051 (3)	0.060 (3)	−0.005 (2)	−0.015 (2)	−0.031 (2)
O3	0.097 (3)	0.085 (3)	0.081 (3)	0.003 (2)	−0.045 (2)	−0.036 (2)
F1	0.20 (2)	0.049 (5)	0.082 (5)	0.045 (7)	−0.079 (9)	−0.028 (4)
F2	0.21 (2)	0.068 (5)	0.084 (6)	−0.002 (6)	−0.088 (10)	−0.037 (4)
F3	0.094 (7)	0.26 (3)	0.055 (5)	−0.013 (14)	0.003 (5)	−0.021 (12)
F1'	0.094 (11)	0.33 (4)	0.063 (7)	0.00 (2)	−0.033 (7)	−0.02 (2)
F2'	0.19 (3)	0.076 (8)	0.065 (7)	0.019 (9)	−0.025 (10)	−0.044 (6)
F3'	0.18 (2)	0.066 (7)	0.063 (7)	−0.045 (9)	−0.021 (9)	−0.015 (5)
C2	0.063 (3)	0.041 (3)	0.060 (3)	0.003 (2)	−0.022 (2)	−0.025 (2)
C3	0.065 (3)	0.043 (3)	0.054 (3)	0.003 (2)	−0.024 (2)	−0.023 (2)
C8	0.058 (3)	0.048 (3)	0.055 (3)	0.001 (2)	−0.022 (2)	−0.025 (2)
C7	0.079 (4)	0.052 (3)	0.071 (3)	0.000 (2)	−0.025 (3)	−0.024 (3)
C5	0.087 (4)	0.093 (4)	0.051 (3)	0.005 (3)	−0.026 (3)	−0.028 (3)
C6	0.104 (5)	0.059 (3)	0.066 (3)	0.000 (3)	−0.040 (3)	−0.016 (3)
C1	0.093 (5)	0.053 (4)	0.059 (4)	0.003 (3)	−0.028 (4)	−0.029 (3)
C4	0.082 (4)	0.075 (4)	0.068 (3)	0.002 (3)	−0.025 (3)	−0.044 (3)

### Geometric parameters (Å, º)

**Table:**

N2—C2	1.314 (5)	F2'—C1	1.303 (13)
N2—C8	1.388 (5)	F3'—C1	1.309 (15)
N2—H2A	0.8600	C2—C1	1.504 (6)
O2—N1	1.260 (4)	C3—C8	1.371 (6)
O1—N1	1.228 (5)	C3—C4	1.402 (6)
N3—C2	1.333 (5)	C8—C7	1.388 (6)
N3—C3	1.384 (5)	C7—C6	1.346 (7)
N3—H3A	0.8600	C7—H7	0.9300
N1—O3	1.226 (5)	C5—C4	1.377 (7)
F1—C1	1.295 (12)	C5—C6	1.393 (8)
F2—C1	1.297 (12)	C5—H5	0.9300
F3—C1	1.240 (10)	C6—H6	0.9300
F1'—C1	1.206 (14)	C4—H4	0.9300
			
C2—N2—C8	108.3 (3)	C5—C6—H6	119.1
C2—N2—H2A	125.9	F1'—C1—F3	132.5 (9)
C8—N2—H2A	125.9	F1'—C1—F1	48 (2)
C2—N3—C3	107.4 (3)	F3—C1—F1	112.0 (13)
C2—N3—H3A	126.3	F1'—C1—F2	55 (2)
C3—N3—H3A	126.3	F3—C1—F2	110.2 (11)
O3—N1—O1	123.2 (4)	F1—C1—F2	102.3 (10)
O3—N1—O2	119.6 (4)	F1'—C1—F2'	108.7 (16)
O1—N1—O2	117.2 (4)	F3—C1—F2'	54.6 (9)
N2—C2—N3	110.6 (4)	F1—C1—F2'	139.1 (9)
N2—C2—C1	125.0 (4)	F2—C1—F2'	59.5 (9)
N3—C2—C1	124.3 (4)	F1'—C1—F3'	110.3 (19)
C8—C3—N3	107.3 (4)	F3—C1—F3'	49.3 (11)
C8—C3—C4	122.1 (4)	F1—C1—F3'	67.9 (9)
N3—C3—C4	130.6 (4)	F2—C1—F3'	141.3 (8)
C3—C8—N2	106.5 (4)	F2'—C1—F3'	102.4 (12)
C3—C8—C7	121.4 (4)	F1'—C1—C2	115.1 (8)
N2—C8—C7	132.1 (4)	F3—C1—C2	112.3 (7)
C6—C7—C8	117.2 (5)	F1—C1—C2	110.6 (6)
C6—C7—H7	121.4	F2—C1—C2	108.9 (6)
C8—C7—H7	121.4	F2'—C1—C2	110.0 (7)
C4—C5—C6	122.3 (5)	F3'—C1—C2	109.6 (7)
C4—C5—H5	118.9	C5—C4—C3	115.1 (5)
C6—C5—H5	118.9	C5—C4—H4	122.4
C7—C6—C5	121.8 (5)	C3—C4—H4	122.4
C7—C6—H6	119.1		
			
C8—N2—C2—N3	0.0 (5)	N2—C2—C1—F1'	−92 (3)
C8—N2—C2—C1	−178.0 (5)	N3—C2—C1—F1'	90 (3)
C3—N3—C2—N2	−0.5 (5)	N2—C2—C1—F3	90 (2)
C3—N3—C2—C1	177.6 (5)	N3—C2—C1—F3	−88 (2)
C2—N3—C3—C8	0.7 (5)	N2—C2—C1—F1	−144.3 (11)
C2—N3—C3—C4	−178.9 (5)	N3—C2—C1—F1	38.0 (13)
N3—C3—C8—N2	−0.7 (5)	N2—C2—C1—F2	−32.6 (16)
C4—C3—C8—N2	179.0 (4)	N3—C2—C1—F2	149.7 (13)
N3—C3—C8—C7	−179.3 (4)	N2—C2—C1—F2'	31 (2)
C4—C3—C8—C7	0.4 (7)	N3—C2—C1—F2'	−146.9 (18)
C2—N2—C8—C3	0.4 (5)	N2—C2—C1—F3'	142.8 (16)
C2—N2—C8—C7	178.8 (5)	N3—C2—C1—F3'	−35.0 (17)
C3—C8—C7—C6	−0.7 (8)	C6—C5—C4—C3	−1.2 (8)
N2—C8—C7—C6	−178.8 (5)	C8—C3—C4—C5	0.5 (7)
C8—C7—C6—C5	0.0 (8)	N3—C3—C4—C5	−179.8 (5)
C4—C5—C6—C7	1.0 (9)		

### Hydrogen-bond geometry (Å, º)

**Table:**

*D*—H···*A*	*D*—H	H···*A*	*D*···*A*	*D*—H···*A*
N2—H2*A*···O1	0.86	1.86	2.703 (5)	168
N3—H3*A*···O2^i^	0.86	1.84	2.682 (5)	165

Symmetry code: (i) *x*, *y*−1, *z*.
